# Supplementary material for: The interactome of intact mitochondria by cross-linking mass spectrometry provides evidence for coexisting respiratory supercomplexes
Source: Mol Cell Proteomics. 2017 Dec 8;17(2):216–32. doi: 10.1074/mcp.RA117.000470 (PMC5795388; doi:10.1074/mcp.RA117.000470)
Supplement: Supplemental Data [file supp_RA117.000470_133922_0_supp_23978_fzffwf.zip › spectra_annotation/mito_DR_spectra_annotation/173-1-10-1-12-1.pdf]

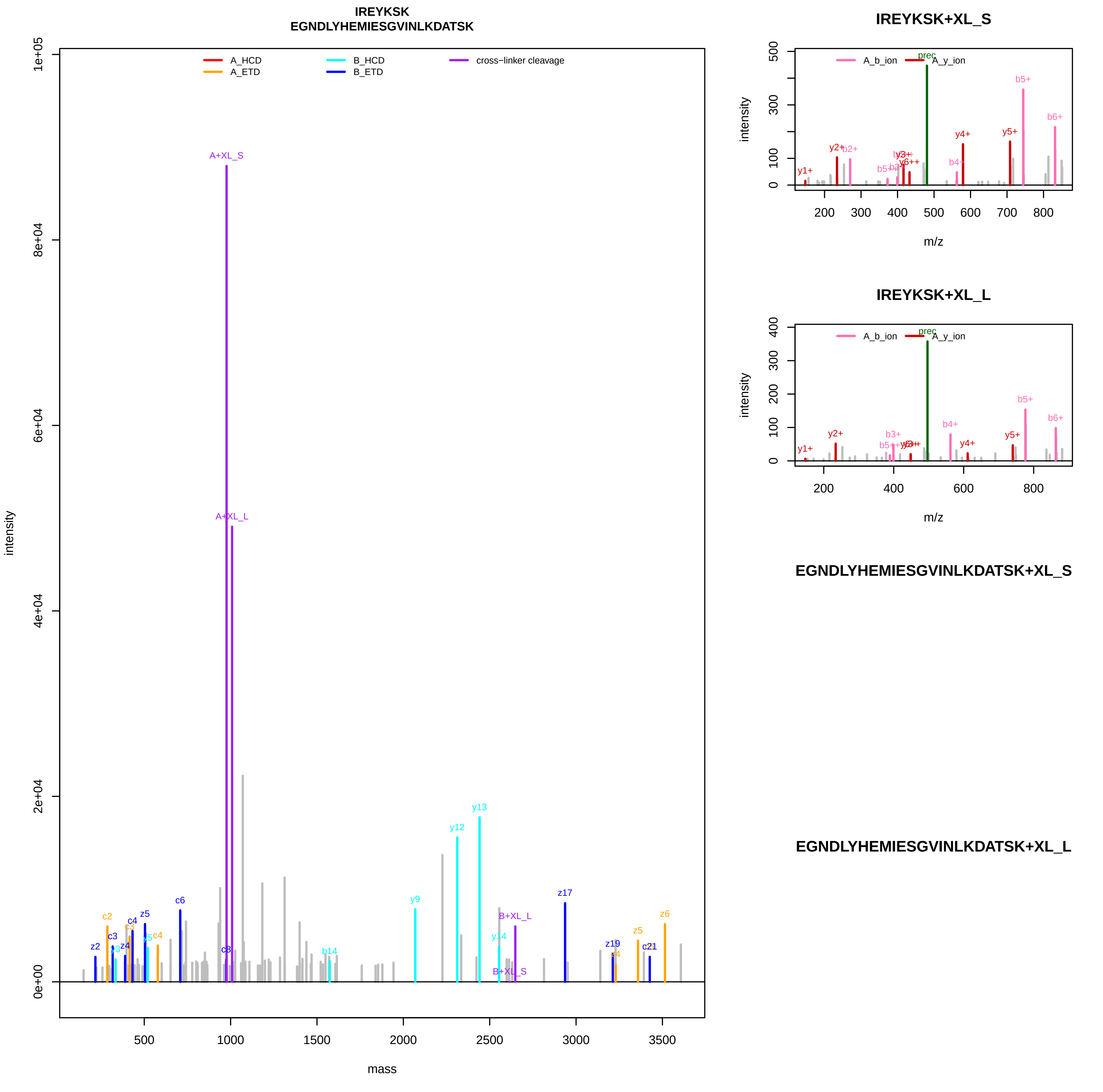
IREYKSK
EGNDLYHEMIESGVINLKDATSK
A_HCD
A_ETD
B_HCD
B_ETD
cross−linker cleavage
A+XL_S
A+XL_L
B+XL_L
B+XL_S
y9
y12
y13
y14
b14
z17
z19
c21
z2
c2
c3
z3
c4
z4
z5
y5
c6
c8
z5
z6
intensity
mass
0e+00
2e+04
4e+04
6e+04
8e+04
1e+05
500
1000
1500
2000
2500
3000
3500
IREYKSK+XL_S
A_b_ion
A_y_ion
prec
y1+
y2+
b2+
y3+
y4+
y5+
y6++
b4+
b5+
b6+
intensity
m/z
0
100
300
500
200
300
400
500
600
700
800
IREYKSK+XL_L
A_b_ion
A_y_ion
prec
y1+
y2+
b3+
y4+
y5+
b4+
b5+
b6+
intensity
m/z
0
100
200
300
400
200
400
600
800
EGNDLYHEMIESGVINLKDATSK+XL_S
EGNDLYHEMIESGVINLKDATSK+XL_L
